# Inpatient post-COVID-19 rehabilitation program featuring virtual reality—Preliminary results of randomized controlled trial

**DOI:** 10.3389/fpubh.2023.1121554

**Published:** 2023-02-06

**Authors:** Sebastian Rutkowski, Katarzyna Bogacz, Anna Rutkowska, Jan Szczegielniak, Richard Casaburi

**Affiliations:** ^1^Faculty of Physical Education and Physiotherapy, Opole University of Technology, Opole, Poland; ^2^Specialist Hospital of the Ministry of the Interior and Administration in Głuchołazy, Głuchołazy, Poland; ^3^Rehabilitation Clinical Trials Center, Lundquist Institute for Biomedical Innovation at Harbor-UCLA Medical Center, Torrance, CA, United States

**Keywords:** COVID-19, pulmonary rehabilitation, post-COVID-19, virtual rehabilitation, virtual reality, post-acute sequelae of COVID-19 (PASC)

## Abstract

**Background:**

Numerous recommendations from pulmonary scientific societies indicate the need to implement rehabilitation programs for patients after COVID-19. The aim of this study was to propose an innovative comprehensive intervention based on a hospital-based pulmonary rehabilitation program for individuals with post-acute sequelae of COVID-19.

**Methods:**

It was decided to evaluate two forms of hospital rehabilitation: traditional and one provided through virtual reality. Preliminary results are based on a group of 32 patients (20 female and 12 male), of average age 57.8 (4.92) years in the period of 3–6 months after the initial infection. Primary outcomes included analysis of lung function, exercise performance and stress level. A 3-week, high-intensity, five-times per week pulmonary rehabilitation program was designed to compare the effectiveness of a traditional form with a VR-led, novel form of therapy.

**Results:**

The analysis of the results showed a statistically significant improvement in both groups with regard to exercise performance expressed as 6MWT distance. Moreover, a statistically significant decrease in dyspnoea levels following the 6MWT was also noted in intergroup comparison, but the between-group comparison revealed non-statistically significant changes with low effect size. Regarding lung function, the analysis showed essentially normal lung function at baseline and a non-statistically significant improvement after the completion of the rehabilitation program. The analysis of the stress level showed a statistically significant improvement in both groups within the inter-group comparison, yet the between-group comparison of deltas values showed a non-significant difference with low effect size.

**Conclusion:**

A 3-weeks inpatients pulmonary rehabilitation program led to improvement of the exercise performance of people with post-acute sequelae of COVID-19, but not lung function. Furthermore, the program was shown to reduce patients' stress levels. A comparison of the traditional form of rehabilitation to the novel form using VR, shows similar effectiveness in terms of exercise performance and stress levels.

## Introduction

Over recent years, SARS-CoV-2 infection has been confirmed in millions of people around the world. The virus spreads mainly through the respiratory tract (especially from droplets arising from coughing, sneezing, and talking) and through contaminated surfaces and biological substances (1). Clinical symptoms of patients with acute COVID-19 infection include fever, sore throat, coughing, fatigue, and gastrointestinal symptoms. In more severe cases, respiratory failure, as well as heart and kidney damage, may occur. This occurs especially in the elderly and in people with concomitant chronic diseases (2). In one report, at about 60 days after onset of the first post-COVID-19 symptom, only 13% of the patients previously hospitalized for COVID-19 were completely free of any COVID-19-related symptoms, while 32% had one or two symptoms and 55% had three or more symptoms (3). A meta-analysis incorporating 18 studies reported 1-year follow-up data from 8,591 COVID-19 survivors; the eight most common symptoms were: fatigue/weakness (28%), dyspnoea (18%), arthromyalgia (26%), depression (23%), anxiety (22%), memory loss (19%), concentration difficulties (18%), and insomnia (12%).

In the initial phase of the epidemic, very few people were diagnosed. Those who were hospitalized had health-threatening “severe” symptoms. In addition to the hospitalized patients with “severe” COVID-19, millions of people have most probably been infected with SARS-CoV-2 without formal COVID-19 testing and/or medical treatment in the hospital (4, 5). These patients are classified as having “mild” COVID-19, as they only require home care and the infection is expected to resolve (6). However, patients with the so-called “mild” COVID-19 may still complain about persistent symptoms, many weeks after the onset of symptoms. A study by Goërtz et al. assessed multiple relevant symptoms in hospitalized and non-hospitalized patients with post-acute sequelae of COVID-19 (PASC) (7). A total of 2,113 patients with persistent complaints after COVID-19 infection were assessed for demographics, pre-existing comorbidities, health status, date of the onset of symptoms, and COVID-19 diagnosis. Patients reported a median number of 14 (IQR 11–17) symptoms, and 97% of the respondents had more than five symptoms. The number of symptoms during the acute infection reduced significantly over 3 months' time: median 14 (IQR 11–17) vs. 6 (IQR 4–9); *p* < 0.001. Fatigue and dyspnoea were the most prevalent symptoms during the acute infection and at follow-up (fatigue: 95 vs. 87%; dyspnoea: 90 vs. 71%). This suggests the presence of a post-acute sequelae of COVID-19, and highlights the unmet healthcare needs in a subgroup of patients with both “mild” and “severe” COVID-19. Moreover, survivors of severe COVID-19 are significantly impaired in all activities of daily living and are in need of multimodal rehabilitation, with particular focus on the cardiovascular and pulmonary systems. It is anticipated that some patients with COVID-19 will have a need for rehabilitation interventions during and immediately after hospitalization (8, 9). However, data on the efficacy of rehabilitation during and/or after hospitalization in these patients are lacking (10).

The benefits of respiratory rehabilitation are well-known and existing programmes can be used as one of the referral paths for the rehabilitation of COVID-19 survivors with symptoms and/or impairment of physical functions. Many systematic literature reviews show the beneficial effect of pulmonary rehabilitation in patients with chronic respiratory diseases on exercise capacity (11), respiratory muscle strength (12), and quality of life (13). Therefore, it has been assumed that the mechanisms leading to improvement of the condition in pulmonary rehabilitation may be beneficial for patients with PASC who present with persistent symptoms and impairment of physical function, especially those with symptoms related to lung function, such as fatigue and dyspnoea. However, data on the efficacy of rehabilitation specifically for PASC patients are lacking. Furthermore, this presumption has been confirmed in one of the world's first randomized trials of patients after a SARS-CoV-2 infection, which showed that a 6-week pulmonary rehabilitation programme improved lung function. The intervention group and control group were compared after 6 weeks of respiratory rehabilitation, and it was found that there was a statistically significant difference in improvement in FEV_1_ (L), FVC (L), and FEV_1_/FVC% (14). Furthermore, The Convergence of Opinion on Recommendations and Evidence process was used to make interim recommendations for in-hospital and post-hospital phase rehabilitation patients with COVID-19 and post-acute sequelae of COVID-19, respectively. The International Task Force (ITS) was established, including the European Respiratory Society (ERS) and the American Thoracic Society (ATS), as well as experts in the field of pulmonary rehabilitation. In its report, the ITS identified recommendations for the rehabilitation of patients with COVID-19 and PASC. The ITS provided recommendations for rehabilitation procedures during the hospitalization and recommendations to multidimensional rehabilitation and functional assessment methods within 6–8 weeks after discharge from the hospital (10).

The use of virtual reality (VR) in pulmonary rehabilitation programs has been reported for several years in the research literature. VR presents engaging scenarios that can shift attention, distracting the patient from negative sensations (e.g., fatigue and shortness of breath) during physical activity (15), and it has also been shown to motivate patients to be active (16). The evaluation of the effectiveness of such technological solutions has benefits in terms of improving physical fitness (17), reducing anxiety and depressive symptoms (18), as well as exercise capacity (19–21).

The aim of this study was to propose an innovative comprehensive intervention based on a pulmonary rehabilitation programme for patients with post-acute sequelae of COVID-19. Moreover, this project evaluates the use of VR in the rehabilitation processes. This study aims to address the following hypotheses:

1. Participation in the 3-week pulmonary rehabilitation programme will improve the pulmonary function and exercise capacity of individuals with post-acute sequelae of COVID-19.
2. Participation in the 3-week pulmonary rehabilitation programme will improve the stress level of individuals post-acute sequelae of COVID-19.

## Materials and methods

### Participants

The study was conducted among patients with PASC who participated in inpatient pulmonary rehabilitation at the Specialist Hospital in Glucholazy (Poland). These preliminary results included 32 randomly selected patients aged 41–67 years old who met the inclusion criteria and gave written consent to participate in the study. The patients were randomized to two groups: experimental (VR group) which participated in the pulmonary rehabilitation program incorporated with VR-based features, and the control group (control group) participating in the same therapeutic elements but in a traditional manner. Randomization was performed using the Research Randomizer (ratio 1:1), a web-based service that offers instant random assignment. Sealed envelopes were used for group assignment. The inclusion criteria were: women and men aged 40–80 years and a confirmation from a primary care physician of having had COVID-19 infection. The exclusion criteria were: no consent to participate, active pneumonia diagnosed by x-ray, documented heart disease (stable or unstable), status after coronary artery bypass grafting, percutaneous transluminal coronary angioplasty, insulin-dependent diabetes mellitus, inability to exercise independently or musculoskeletal/neurological conditions that would prevent completion of the course, lung cancer, cognitive impairment, or Mini-Mental State Examination < 24. This study implemented a randomized control trial study design, approved by Bioethical commission Opole Medical Chamber in Opole (Approval Number: No. 343, 25 November 2021), registered in ClinicalTrials.gov (NCT05244135), and carried out in accordance with the Declaration of Helsinki guidelines (22). All participants gave written informed consent.

### Intervention

A 3-week, five-times-week high-intensity pulmonary rehabilitation program was designed to compare the effectiveness of a traditional form with a VR-led, novel form of therapy. Such programs have been shown to produce clinically significant improvements in exercise capacity, dyspnea, quality of life and lung function in patients with COPD (23, 24) or lung cancer (25). The program was based on previous experience in patients with COPD (26) and employed a holistic bio-psycho-social approach for SARS-CoV-2 patients with combined treatment aimed at increasing exercise capacity, restoring lung function and supporting mental health and was delivered by a multidisciplinary team. A detailed description of the rehabilitation program has been described previously (27). The program addressed patient eligibility for one of five rehabilitation models varying in therapy intensity. Qualification for each model included the result of a 6-minute walk test and an assessment of the level of dyspnea (modified Borg Dyspnoea Scale; Table 1). All models included the same components: exercise training on the cycle ergometer, breathing exercises, general fitness exercises, resistance training, and relaxation.

**Table 1:** Qualification criteria for pulmonary rehabilitation model.

**Borg**	**6-minute walk distance**			
**dyspnea rating**	<**320 m**	**320–434 m**	**435–520 m**	>**520 m**
8–7	Model D	Model D	Model C	Model B
6–4	Model D	Model D or C	Model C or B	Model B or A
3–2	Model D	Model C	Model B	Model A
0–1	Model D	Model C	Model B	Model A

Cycle ergometer training was conducted at the training heart rate—which was a percentage of the achieved heart rate in the 6-minute walk test. The limits of the initial training heart rate on the cycle ergometer, varied depending on the model (model A-−90% of peak 6-minute walk heart rate, model B-−80%, model C-−70%, and model D-−20–30% increase in heart rate during exercise compared to resting heart rate). Initial work rate and increment were also adapted to the rehabilitation model:

- Model A−50W, 70W, 90W, 110W, 130W, 150W
- Model B−50W, 60W, 70W, 80W, 90W, 100W
- Model C−30W, 40W, 50W, 60W, 70W, 80W
- Model D−30W

Moreover, the Virtual Park software features an automatic system of changes in training heart rate: after reaching the target heart rate in the previous training session, the work rate on the last phase was increased by 5W; if the heart rate was not reached, the final work rate was reduced by 5W. Prior to training, the therapist recorded heart rate (HR) and pulse oximeter saturation (SpO_2_) values, dyspnea and fatigue (rated on a modified Borg scale of 1–10). The therapist was able to see HR and SpO_2_, cycling speed and virtual distance traveled information on a laptop monitor. Cycling cadence was maintained in the 50–70 RPM range. In the control group, patients participated in exercise training on a cycle ergometer without additional audio-visual stimuli. Patients in the VR group participated in the training on the same equipment, however, in addition, a head-mounted display (VR goggles) was employed during the training. Training on the cycle ergometer was held twice a day, while the remaining elements were conducted once a day for both groups.

With regard to relaxation training, patients in the control group participated in Schulz autogenic training while patients in the VR group were given guided relaxation in a virtual setting using the goggles. The time components were the same in both groups.

### Exercise training in VR

**Figure 1:**
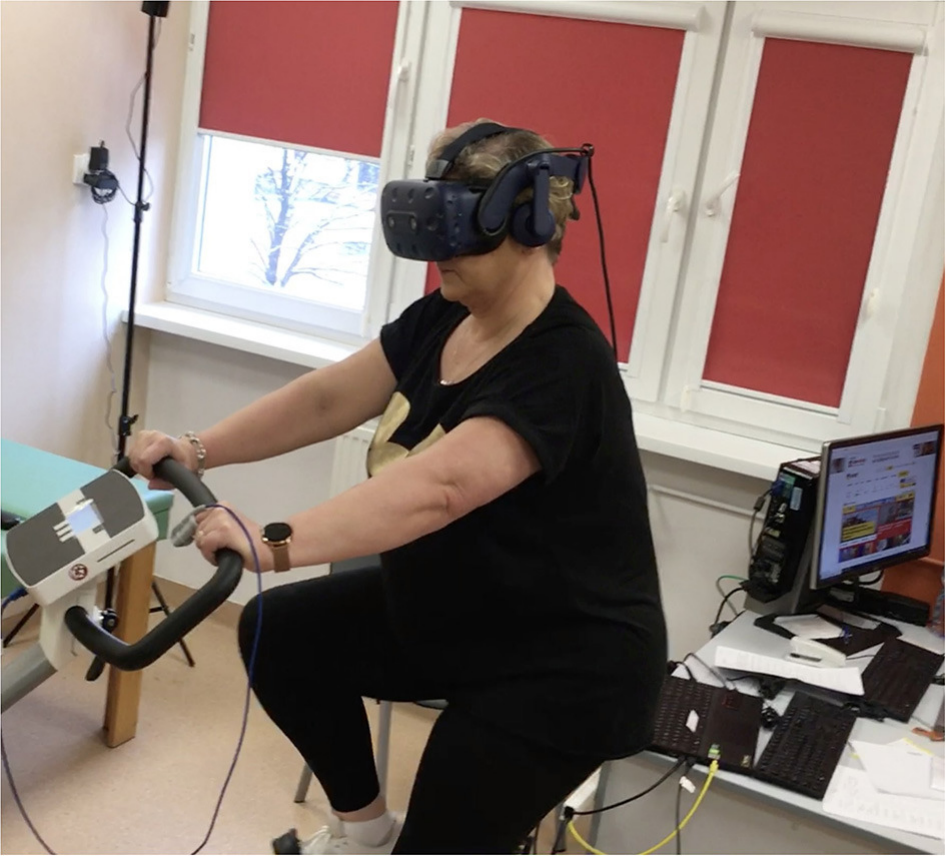
Exercise training on cycle ergometer with VR.

The VR group conducted endurance training using the “Virtual Park” software developed by STIIMA-CNR. The scenery depicted a sunny island where the participants conducted a bicycle ride enriched with realistic elements and sound effects to simulate real-life situations. The software was linked to the ergometer so the dynamics of the image changed with the cadence of pedaling. The training station consisted of a COSMED cycle ergometer, VR goggles, and physiological sensors—an HR band and pulse oximeter (Figure 1). The system had previously been used with patients with respiratory disorders (15).

### Relaxation in virtual reality

**Figure 2:**
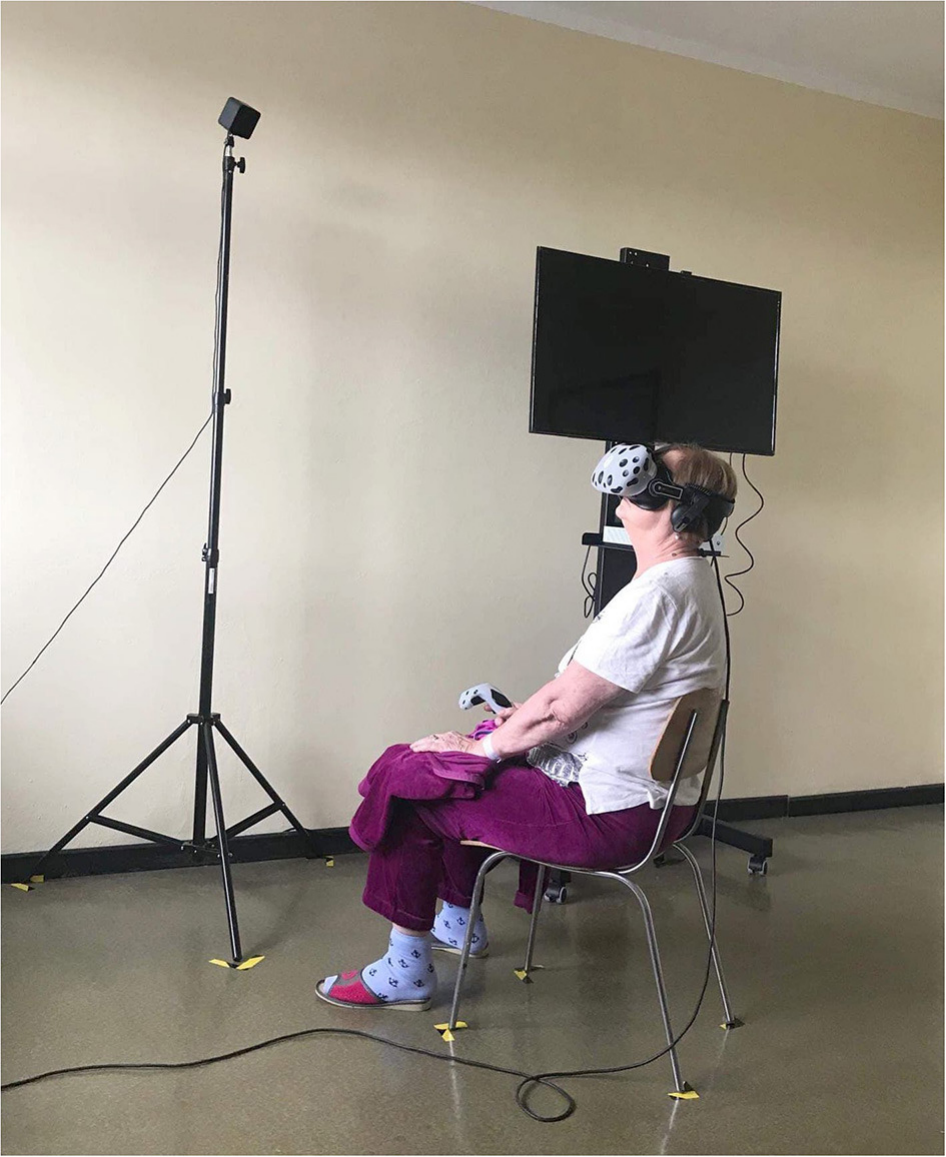
VR station for relaxation.

A VR TierOne device (Stolgraf^®^, Stanowice, Poland) was used as the VR source. The workstation consisted of head-mounted display goggles connected to a computer and a chair on which relaxation was conducted (Figure 2). The software is designed to introduce calmness, mood enhancement and motivation of patients for rehabilitation. The software is based on the Ericsson psychotherapy approach and represents a virtual therapeutic garden. The metaphor of a garden was used to illustrate the patient's condition: at the beginning of rehabilitation, it appears disordered and gray, but with each session it becomes more colorful and lively, symbolizing the process of regaining energy and vigor. Previous studies have shown that the device incorporated into the rehabilitation process improves symptoms of deconditioning, anxiety and stress in pulmonary patients (18).

### Measurement

At baseline, participants completed a self-administered sociodemographic questionnaire. Questions included gender, marital status, place of residence, education, comorbidities, and history of hospitalization for SARS-CoV2 infection.

### Functional capacity

The functional capacity assessment included exercise performance (6-minute walk test: 6MWT), spirometry [forced expiratory volume for 1 second (FEV_1_), forced vital capacity (FVC), and FEV_1_/VC], and plethysmography [total lung capacity (TLC)]. The 6-minute walk test, which is a reliable measure of exercise performance (28) was performed over a length of 30 m; the test result was the distance walked. Patients were instructed to walk as long a distance as possible, allowing them to move independently and rest if necessary. The subjective level of perceived fatigue and dyspnea on the Borg scales at the competition of the test were noted. The tests were conducted in accordance with European Respiratory Society/American Thoracic Society guidelines (29).

### Stress level

The Perceived Stress Scale (PSS-10) is a widely used psychological instrument for measuring the perception of stress (30). It is a measure of the degree to which situations in one's life are appraised as stressful. Items were designed to tap into how unpredictable, uncontrollable, and overloaded respondents find their lives. The scale also includes a number of direct queries about current levels of experienced stress. A lower score implies less impairment.

These examinations were performed before the start of rehabilitation and after its completion.

### Statistical analysis

Analyses were performed using Statistica 13 software (StatSoft, Cracow, Poland) and JASP software (JASP Team, Amsterdam, Netherlands) (31). The statistical significance level was set at α = 0.05. Categorical variables were presented as numeric values and percentages. Continuous variables were presented as mean ± standard deviation (SD) or median and interquartile range [IQR]. Differences in pre-post rehabilitation values were analyzed by paired t-student test or Wilcoxon test, depending on the distribution of the variables. Evaluation of between-group differences was assessed using the Mann-Whitney *U*-test. Multiple linear regression (stepwise) was used to determine whether baseline stress was a determinant of 6MWT improvement in the two study groups. Prior to the multiple linear regression analysis, the assumption of a linear relationship (using the point biserial correlation coefficient) between the outcome variable and the independent variables was tested. The sample size was calculated based on a previous randomized controlled trial (RCT) investigating the effectiveness of inpatient pulmonary rehabilitation in COVID-19 patients which demonstrated an effect size of −0.713 (14) for 6MWT distance change. Due to the length of the reference RCT (6 weeks), it was decided to assume half of this effect size (0.356). G^*^Power 3.1 software was used to calculate the sample size. Calculation was based on repeated-measures ANOVA: the within-between interaction type I error rate was set at 5% (α = 0.05), the effect size of the main outcome was set at 0.356 and the type II error rate gave 95% power for the two groups and two repeated sets of measurements; correlation among the repeated measures was assumed at 0.5 and the non-sphericity correction ε was 1.0. Based on these assumptions, it was determined that 28 patients should be enrolled. The effect sizes were calculated with Cohen's *d*. An effect size ≥ 0.20 was considered small, while an effect size ≥ 0.50 was considered medium and an effect size ≥ 0.80 was considered large (32).

## Results

The main groups characteristic is presented in Table 2.

**Table 2:** Groups characteristic.

	**Total** **(*n* = 32)**	**VR group** **(*n* = 18)**	**Control group** **(*n* = 14)**	***p***
**Gender**, ***n*** **(%)**				
Female	20 (68.75)	11 (61.11)	9 (64.29)	0.860
Male	12 (37.5)	7 (38.88)	5 (35.71)	
**Age**, years, mean (*SD*)	57.8 (4.92)	59.22 (3.89)	56.00 (6.57)	0.413
**Education**, ***n*** **(%)**				
Basic/vocational	6 (18.75)	3 (16.66)	3 (21.43)	0.473
Secondary	12 (37.50)	6 (33.33)	6 (42.86)	
Higher education	14 (43.75)	9 (50.00)	5 (35.71)	
**Current employment status**, ***n*** **(%)**				
Professionally active	21 (65.25)	10 (55.56)	11 (78.57)	0.188
Retirement	11 (34.75)	8 (44.44)	3 (21.43)	
**Marital status**, ***n*** **(%)**				
Married	27 (84.37)	16 (88.88)	9 (64.29)	0.511
Single	1 (3.12)	0 (0.0)	1 (7.14)	
Divorced	4 (1.50)	1 (5.55)	3 (21.43)	
Widow	2 (6.25)	1 (5.55)	1 (7.14)	
**COVID-19 treatment**, ***n*** **(%)**				
Home	21 (65.63)	10 (55.56)	11 (78.57)	0.188
Hospital	11 (34.37)	8 (44.44)	3 (21.43)	

**Figure 3:**
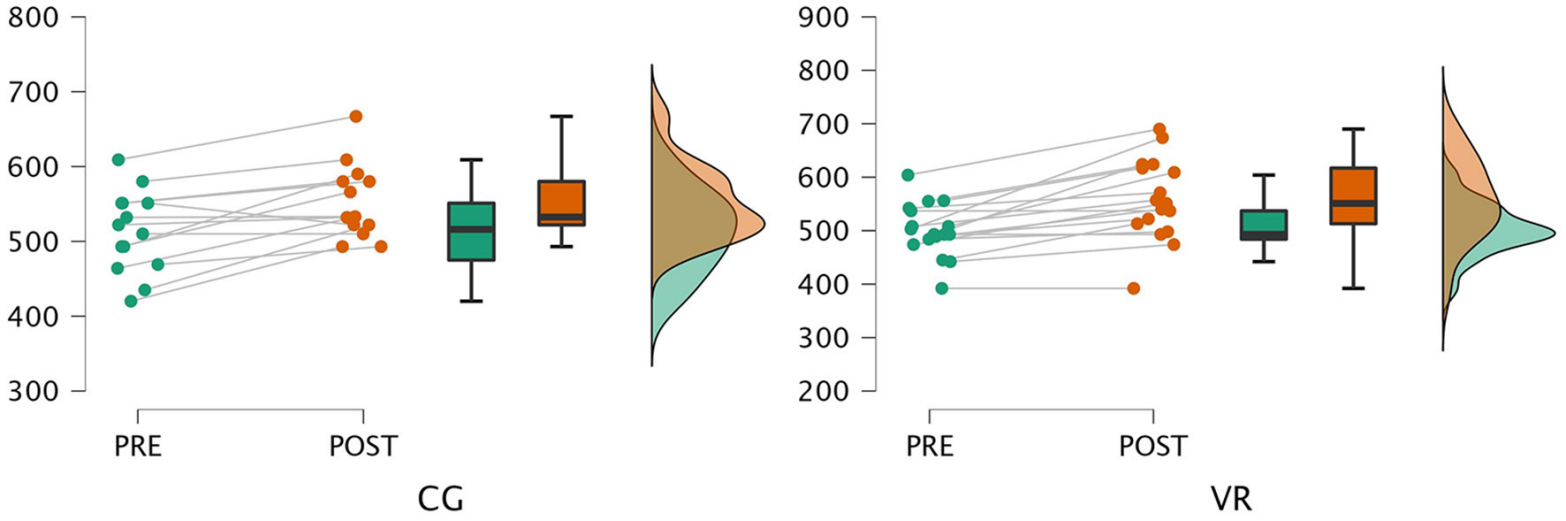
Results of the 6-minute walk test.

The analysis of the results showed a statistically significant improvement in both groups with regard to exercise performance expressed as 6MWT distance (CG: *p* < 0.001; VR: *p* < 0.001). Both groups achieved an average improvement above minimal clinically important difference for 6MWT (56.9 vs. 39.2 in the VR and control groups, respectively). The between-group comparison revealed a non-significant change with a small effect size (Figure 3). Moreover, a statistically significant decrease in dyspnoea levels following the 6MWT was also noted in intergroup comparison (CG: *p* < 0.004, VR: *p* < 0.033), but the between-group comparison revealed non-statistically significant changes with low effect size.

Regarding lung function, the analysis showed essentially normal lung function at baseline and a non-statistically significant improvement after the completion of the rehabilitation program.

The analysis of the stress level showed a statistically significant improvement in both groups (CG: *p* < 0.004; VR: *p* < 0.015) within the inter-group comparison, yet the between-group comparison of deltas values showed a non-significant difference with low effect size (Table 3).

**Table 3:** Results of the analyzed outcomes.

**Variables**	**VR group (*****n*** = **18)**			**Control group (*****n*** = **14)**			**Between group comparison**	
	**Pre**	**Post**	***p***	**Pre**	**Post**	***p***	***p***	**Effect size d**
**6-minute walk test**								
Fatigue	3.0 [1.0]	2.0 [1.0]	0.055	3.0 [1.0]	2.0 [1.0]	**0.010**	0.523	0.233
Dyspnea	2.0 [3.0]	0.0 [2.0]	**0.033**	2.0 [2.75]	0.5 [2.0]	**0.004**	0.621	0.180
Distance (m)	502 (48.4)	558 (76)	**< 0.001**	512 (54.3)	552 (49.1)	**< 0.001**	0.264	−0.411
**Lung function**								
FEV_1_ *(l)*	2.8 (0.66)	2.77 (0.68)	0.708	2.72 (0.60)	2.76 (0.61)	0.879	0.267	0.403
FEV_1_ *(%pred)*	96.6 (13.3)	96.1 (13.1)	0.738	96.9 (17.5)	97.8 (14.1)	1.000	0.599	0.192
FVC *(l)*	3.46 (0.78)	3.56 (0.8)	0.087	3.49 (0.86)	3.5 (0.85)	0.168	0.673	0.152
FVC *(%pred)*	94.1 (11.8)	97.1 (9.0)	0.093	98 (19.6)	98.1 (16.3)	0.233	0.276	−0.401
FEV_1_%VC	84.3 [6.89]	81.4 [7.77]	**0.028**	82.4 [5.32]	83.2 [10.1]	0.130	0.100	0.605
TLC *(l)*	5.18 (1.2)	5.39 (1.1)	0.288	4.9 (1.2)	5.27 (1.0)	0.070	0.403	0.302
**Stress level**								
PSS-10	23.1 (4.2)	20.7 (3.5)	**0.015**	23.8 (3.1)	22.2 (3.8)	**0.004**	0.719	0.080

Bold highlights statistical significance p < 0.05.

FEV1, forced expiratory volume for 1 s; FVC, forced vital capacity; FEV1%VC, forced expiratory volume in one second % of vital capacity; TLC, total lung capacity; % pred, predicted values from Viljanen (33).

Data is presented as mean (SD) or median [IRQ]. Differences in pre-post rehabilitation values were analyzed by paired t-student test or Wilcoxon test, depending on the distribution of the variables. Evaluation of between-group differences was assessed using the Mann-Whitney U-test.

Stepwise multiple regression was used to examine how stress level could explain a statistically significant amount of variance in functional capacity (Table 4). For change in end-exercise dyspnea (difference between the final value and the initial value), baseline PSS-10 was found to be a significant predictor variable, accounting for 13% of the variance in these models (*p* = 0.026). Analysis of the change in 6MWD revealed no correlation with the initial values.

**Table 4:** Stress level (PSS-10) a predictor for functional capacity outcomes (stepwise regression results).

**Variable**	***B***	**Beta**	***t***	***p***	***F***	***R* ^2^**
Change in end-exercise dyspnea	0.171	0.406	2.353	0.026	5.539	0.135

The following covariates were considered but not included: TLC pre, FVC pre, FEV_1_ pre, and FEV_1_%VC pre.

## Discussion

In COVID-19 survivors, physical impairment can persist for weeks after COVID-19, such as shortness of breath, desaturation, cough, weakness, and fatigue. Even in the early stages of the pandemic, expert opinions suggested the need for rehabilitation, based on the fact that exercise is feasible and useful in survivors of critical illness (10). There is not yet a great evidence bases on specific physical rehabilitation for patients with PASC; low to moderate intensity exercise with safety as a priority has been recommended. It was pointed out that the rehabilitation program should take into account needs and functional impairment on an individual basis. Thus, the implemented pulmonary rehabilitation program is in line with earlier recommendations. In order to compare the effectiveness of forms of rehabilitation, it was decided to create two study groups with the same time frame (34). Analysis of the results shows that a 3-week hospital-based rehabilitation program in both forms yields similar results. A clinically relevant observation is that exercise tolerance as assessed by the 6-minute walk test has improved. Moreover, both groups also showed a reduction in dyspnea levels assessed after 6MWT performance. The results also showed that the program did not change spirometric values. However, baseline lung function did not show restrictive or obstructive defects. Thus, it seems that our study group did not manifest residual significant lung function consequences of COVID-19 infection. The third area of analyzed measures was stress level which showed statistically significant improvement in both groups. Moreover, the regression analysis showed that the baseline level of stress influenced some aspects of rehabilitation effectiveness. Thus, it can be preliminarily concluded that rehabilitation conducted in virtual reality yields similar results as the traditional form. Previous in-house experience suggested that, in healthy subjects, virtual reality training in submaximal training generates lower levels of stress (35). There was a greater improvement in the 6MWT distance in the VR group; however, assuming that this element contributed to the improvement should be considered with caution. Similar considerations may apply to stress reduction. In earlier studies with patients with COPD, a VR-based relaxation program was shown to have greater effectiveness compared to guided Schultz audio training (18). It is also planned, once the clinical part of the project is completed, to conduct a technology acceptability analysis to assess satisfaction and sense of presence in VR, which may clarify the mechanism of impact of the virtual reality-based program. The authors felt, though, that it is important to present preliminary data to encourage participation in rehabilitation programs for individuals after COVID-19. The latest meta-analysis included only 3 RCT, where only two studies were conducted in a hospital format, vs. one in tele-rehabilitation form (36).

Our study contributes to the growing literature on the benefits of rehabilitation for patients admitted to inpatient rehabilitation after COVID-19 infection. Previous European studies have shown improvements after inpatient rehabilitation programs in physical fitness, ADL ability, and pulmonary measurements. A study by Piquet et al. evaluated the effectiveness of inpatient rehabilitation for 100 post-acute care COVID-19 patients (37), in which each patient was provided with two (< 20 min) physiotherapy sessions per day. The therapy program included general strengthening of the musculoskeletal system using body weight exercises (sit-to-stand, standing on tiptoes, and squats), rubber bands, and weights. Respiratory training included controlled breathing exercises. Aerobic work included sessions on a bicycle ergometer at submaximal intensity. There was a statistically significant increase in the Barthel Index from admission to discharge, including improvements in the sit-to-stand test and grip strength. Similarly, a study by Puchner et al. evaluated dysfunctions and outcome of COVID-19 survivors after early post-acute rehabilitation (38). These authors showed a significant improvement in lung function, as reflected by an increase of FVC, FEV_1_, TLC, and diffusion capacity for carbon monoxide. A significant improvement in the 6-minute walk test distance was also noted, averaging 176 ± 137 m. Another study evaluating post-acute inpatient rehabilitation was conducted by Curci et al. (39). The program included two sessions of 30 min each day, with average length of stay of 32 days. Forty-one patients were included in the study, where the average length of stay in the rehabilitation unit was 32 ± 9 days. A statistically significant improvement in ADL (Barthel index) and 6MW distance 63 m was observed. Olezene et al. evaluated the effectiveness of inpatient rehabilitation in 29 patients, reporting statistically significant improvements in mobility, cognition, speech, swallowing and balance by the time of discharge (40). Liu et al. implemented a 6-week rehabilitation program which evaluated respiratory function, quality of life, mobility and psychological function in a group of 36 elderly patients with COVID-19 (14). The program significantly improved FEV_1_, FVC, and FEV_1_/FVC%, as well as the 6MW test distance. Thus, it appears that adherence to rehabilitation programs in the sub-acute phase as well as more than 3 months after the infection provides benefits in terms of improving lung function and exercise tolerance. However, studies evaluating the persistence of the effects are lacking. It will be interesting to see whether the results obtained will be seen by only in the following 6–12 months, as in the case of COPD patients, or perhaps longer.

Although this study provides evidence for the effectiveness of hospital-based rehabilitation programs, we recognize that some limitations should be considered. First, the lack of inclusion of data from the full spirometry (including FEF 25–75%) study in the analysis may bias the results. Second, future studies could be enriched with a wider range of diagnostic tools, including more objective ways to measure stress levels (e.g., cortisol levels). Third, a follow-up assessment could provide additional valuable information on efficacy comparisons with traditional therapies. Finally, the subgroup analysis omitted inclusion of the time of symptom onset, which may impact the validity of the results. It is acknowledged that an increase in symptom duration is positively correlated with the magnitude of the immune response and, as such, may influence the recovery of patients. However, we would like to emphasize that the results obtained are preliminary reports, and the project is still in the implementation phase.

## Conclusions

From a clinical perspective, this study's results suggest that VR rehabilitation may be an effective intervention for improving exercise performance and reducing dyspnea levels in patients with post-acute sequelae of COVID-19, although VR was not shown to be more effective than standard rehabilitative practices. This is important as in this patient's cohort are a new population and not much is known about the long-term effects of the virus on lung function, exercise performance and dyspnea levels. The study's findings suggest that VR rehabilitation can help improve exercise performance and reduce dyspnea levels, which can improve patients' quality of life and ability to perform daily activities. Moreover, the results contribute to the understanding of the long-term effects of COVID-19 on the lung and the effectiveness of rehabilitation interventions in patients with post-acute sequelae of COVID-19. Furthermore, the finding that baseline stress level was a significant predictor of changes in end-exercise dyspnea highlights the importance of addressing stress in rehabilitation patients with post-acute sequelae of COVID-19, which is important for the overall wellbeing.

## Data availability statement

The raw data supporting the conclusions of this article will be made available by the authors, without undue reservation.

## Ethics statement

The studies involving human participants were reviewed and approved by Bioethical Commission Opole Medical Chamber in Opole (Approval Number: 343, 25 November 2021). The patients/participants provided their written informed consent to participate in this study.

## Author contributions

SR: conceptualization, project administration, and funding acquisition. SR, JS, and RC: methodology. SR and RC: formal analysis. KB, AR, and JS: investigation. SR, AR, and RC: writing—original draft preparation. RC: supervision. All authors: writing—review and editing, meet criteria for authorship as recommended by the International Committee of Medical Journal Editors (ICMJE), read and approved the final manuscript, and agree to be accountable for all aspects of the work in ensuring that questions related to the accuracy or integrity of any part of the work are appropriately investigated and resolved.
